# Rare presentation of Waldenström’s macroglobulinaemia requiring bilateral above-knee amputations: a case report

**DOI:** 10.1186/s13256-022-03622-1

**Published:** 2022-11-03

**Authors:** Raeed Deen, Calyb Austin, Alexander Bowden, Andrew Bullen

**Affiliations:** 1 Department of Vascular Surgery, Wollongong Hospital, Wollongong, NSW Australia; 2 Department of Haematology, Wollongong Hospital, Wollongong, NSW Australia

**Keywords:** Waldenström’s macroglobulinaemia, Lower limb ischemia, Amputation, Case report

## Abstract

**Background:**

Waldenström’s macroglobulinaemia is a rarely encountered B-lymphocytic malignancy. Waldenström’s macroglobulinaemia-associated paraproteinaemia is linked to an increase in serum viscosity, which results in a hypercoagulable state. Burning bilateral foot pain in a man with alcohol dependence and controlled atrial fibrillation presenting to the emergency department was attributed to peripheral neuropathy, given satisfactory angiographic evidence of bilateral foot arterial blood supply. Subsequently, his presentation as an emergency with acute bilateral critical lower limb ischemia that was managed by bilateral above-knee amputations, prompted a wider search for other etiologies. We present a hitherto unreported case of Waldenström’s macroglobulinaemia-related acute bilateral lower limb ischemia, which required bilateral above-knee amputations.

**Case presentation:**

A 50-year-old Caucasian man, who was an alcohol dependent heavy smoker, presented with burning pain in his right foot that was deemed to be related to alcoholic neuropathy. A computerized tomographic angiogram demonstrated an occluded right distal anterior tibial artery but a patent posterior tibial artery supplying the foot arch, findings that were associated with noncritical ischemia. After multiple presentations within a week, he was admitted following sudden clinical deterioration with acute confusion, hyponatremia, and bilateral foot pain. Over the course of 24 hours, the patient deteriorated rapidly, with bilateral lower limb ischemia requiring bilateral above-knee amputations. Subsequent investigations revealed a diagnosis of Waldenström’s macroglobulinaemia.

**Conclusions:**

To the best of our knowledge, this is the only reported case of Waldenström’s macroglobulinaemia-induced bilateral lower limb ischemia requiring major bilateral amputations.

## Introduction

Waldenström’s macroglobulinaemia (WM) is a rare malignancy of terminally differentiated B lymphocytes characterized by excessive monoclonal production of immunoglobulin M (IgM). It is considered both a lymphoproliferative disorder and a plasma cell dyscrasia, with an incidence of 3 per million a year and 5-year survival rate of 52% [1]. It primarily affects male patients, with a median age at diagnosis of 70 years; it is uncommon in patients less than 50 years old [2]. Its etiology is uncertain, and while the majority are sporadic, somatic mutations in *MYD88* and *CXCR4* have been identified, though these have limited specificity and sensitivity, respectively [3].

WM has a highly variable clinical presentation, with the majority presenting with weakness, fatigue, and weight loss. Approximately 25% of patients are asymptomatic at diagnosis [4]. WM also manifests as symptomatic cytopenia, peripheral neuropathy, hyperviscosity syndrome, or cryoglobulinemia [5, 6], and these may uncommonly be the primary findings at time of diagnosis.

We report a case of atypical presentation of WM with abrupt onset of catastrophic lower limb arterial occlusive symptoms and rapid deterioration, resulting in bilateral above-knee amputation.

## Case presentation

A 50-year-old Caucasian male presented to the emergency department with a 2-month history of “burning” pain in his right foot. He had a background history of paroxysmal atrial fibrillation, Grave’s disease that required partial thyroidectomy, alcohol dependency, and a 30-pack year history of smoking. His atrial fibrillation was managed with metoprolol only, a cardio-selective beta blocker, and his thyroid function was supplemented with levothyroxine. There had been no recent new medications commenced. There was no known prior history of peripheral vascular disease or diabetes.

On examination, his right foot was cool to touch without discoloration or tissue loss. He had a strong posterior tibialis pulse and an absent dorsalis pedis pulse. There was reduced sensation in both feet due to longstanding alcoholic peripheral neuropathy; lower limb motor function was intact. His left lower limb had a full complement of pulses. Doppler assessment by his bedside showed a triphasic signal over the right mid anterior tibial artery, indicating normal flow, but confirmed the absence of arterial flow through the dorsalis pedis artery on the right foot.

**Fig. 1:**
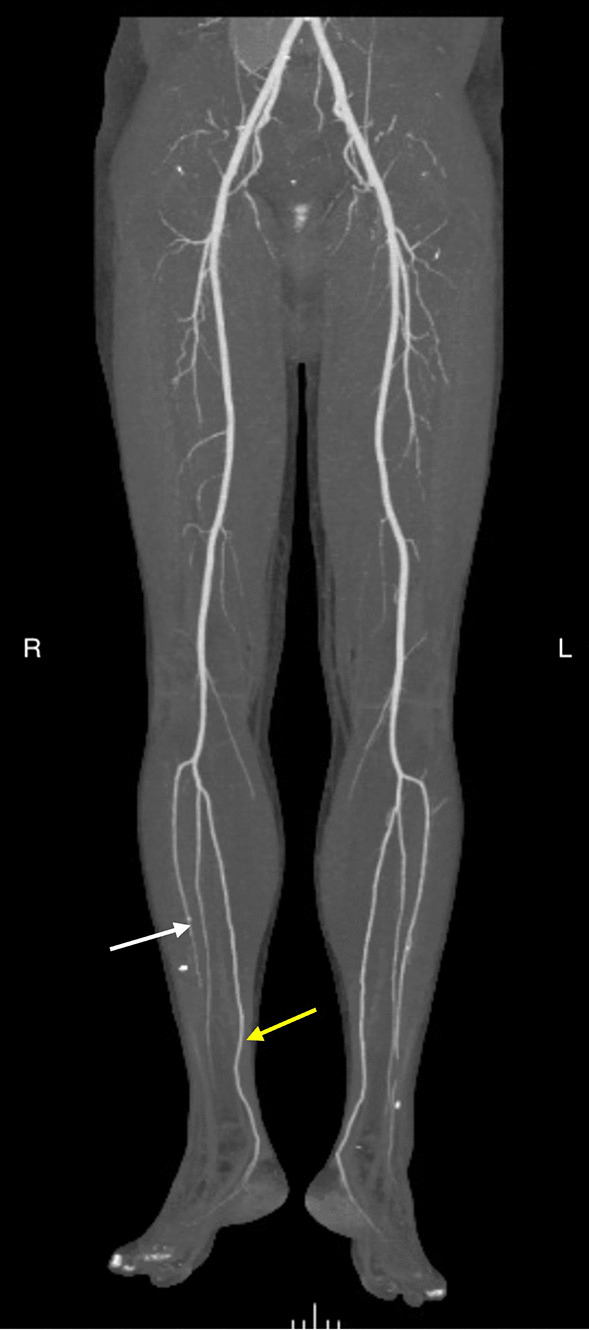
CT-angiography of the lower limbs demonstrating the right anterior tibial artery occluding in the distal leg (white arrow) with a patent right posterior tibial artery (yellow arrow). Note normal arteriogram of the left leg

Computerized tomographic angiography from the arch of the aorta to the lower limbs confirmed the examination findings, demonstrating patent right posterior tibial and peroneal arteries, and a right anterior tibial artery that occluded distally with no flow into the dorsalis pedis artery (Fig. 1). The right foot vascular arch was preserved, with the main inflow being via the posterior tibial artery. There was no large vessel occlusion proximally in the thigh, and no aortoiliac abnormalities. Given the chronicity of the symptoms and investigation findings, his right foot pain was attributed to alcohol-related neuropathy and the patient was discharged from hospital with lifestyle modifying advice. Subsequently, he had presented to the emergency department on several occasions over the following few days with the same complaint. Two consecutive blood investigations including total leukocyte counts, neutrophil percentage, and hemoglobin levels were within normal values, while platelet counts on both occasions were marginally elevated at 549 × 10^9^/L and 440 × 10^9^/L, respectively. Furthermore, blood coagulation profiles, which included activated partial thromboplastin time, prothrombin time, and international normalized ratios were within normal limits on both occasions. With similar examination findings to his previous admissions and normal biochemical markers, he was again discharged with analgesic medication. A week later, he was brought into the emergency department with acute confusion, bilateral foot pain, hyponatremia, and elevated inflammatory markers. This time his total leukocyte count was elevated at 18 × 10^9^/L, hemoglobin concentration was 119 g/L, platelet count was 350 × 10^9^/L, and the hematocrit assay was 0.35/L, which remained unchanged from hematocrit values at previous visits. Likewise, coagulation profiles remained normal. A septic workup, which included a lumbar puncture, did not reveal an obvious infective cause. CT and magnetic resonance imaging (MRI) of the brain were unremarkable. Lower limb venous Doppler assessment was negative for deep venous thromboses. His hyponatremia was appropriately corrected with fluid restriction resulting in resolution of confusion. However, on this occasion, he complained of worsening pain in both feet with a rapid onset of new livedo reticularis of both feet. At presentation, the working diagnosis was of a cardioembolic phenomenon in the context of paroxysmal atrial fibrillation, and he was commenced on therapeutic anticoagulation. Subsequent transesophageal echocardiogram did not show an intramural thrombus or a patent foramen ovale.

**Fig. 2:**
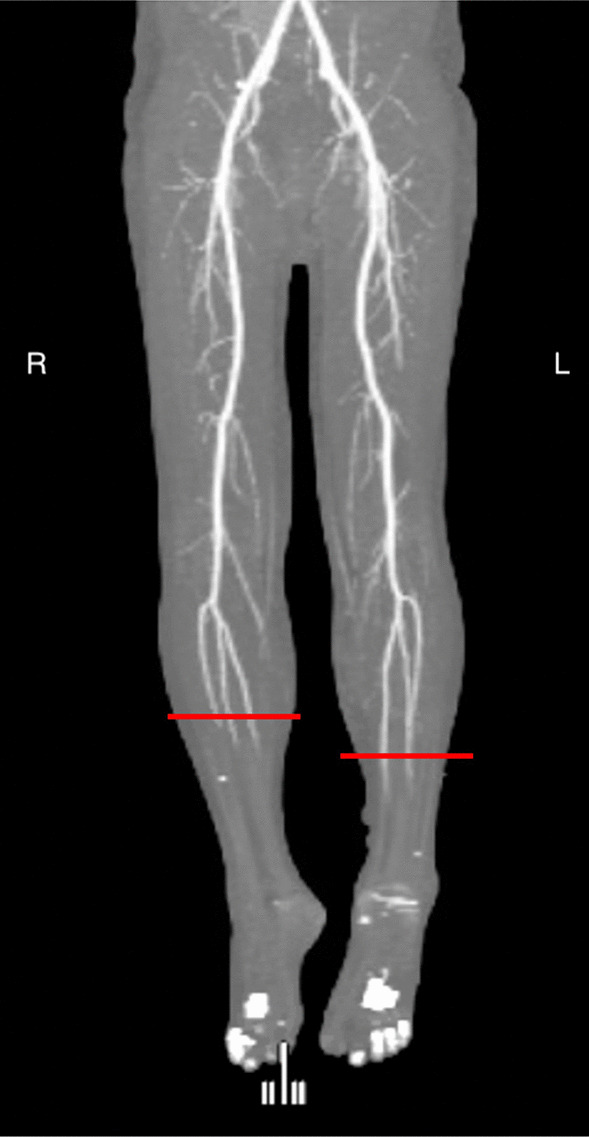
Repeat CT-angiography of the lower limbs (1 week later, during acute admission) demonstrating absent arterial flow past the mid-leg bilaterally. The red lines indicate the point of angiographic “cut-off” of leg blood supply

The following day, the patient’s condition and limb pain worsened; he had “trash feet” and new motor dysfunction of the feet. Overnight, his lower limb ischemia had worsened rapidly to the point where revascularization was no longer a viable option. A repeat CT-angiogram of the lower limbs showed absent arterial flow of contrast bilaterally below the mid-leg, with no evidence of aortic or large vessel pathology (Fig. 2). He was deemed to have non-salvageable Rutherford Grade 3 acute limb ischemia in bilateral lower limbs below the knees. To avoid the complication of multiorgan failure that may have resulted from release of myoglobin, intramuscular potassium, and inflammatory cytokines, he underwent emergent bilateral above-knee amputations, with satisfactory improvement in his clinical status.

A cause for his trash feet was not found prior to the operation that was empirically attributed to cardioembolic pathology. However, the indicator to search for another diagnosis of acute bilateral arterial occlusion of his leg arteries was a positive direct antiglobulin test that indicated the presence of cold agglutinins in his blood. This was performed during blood grouping before major surgical intervention. These findings led to further serological testing, including serum immunofixation electrophoresis. This indicated the presence of a monoclonal B cell population involving 32% of his total lymphocyte count, as well as a mixed IgM Kappa and IgM Lambda cryoglobulinemia, an indication for bone marrow assessment. The diagnosis of Waldenström’s macroglobulinaemia was confirmed with a bone marrow aspirate, which demonstrated an* MYD88*-negative monoclonal B cell population with immunohistochemistry and flow cytometry that was typical for WM. With guidance from the hematology team, he was commenced on a regime of high-dose prednisolone (100 mg/day) and chemoimmunotherapy. His treatment schedule was rituximab (375 mg/m^2^) and bendamustine (90 mg/m^2^) on day 1 of the cycle, and bendamustine (90 mg/m^2^) on day 2, every 30 days for 5 cycles. Response was measured by serum levels of cryoglobulin. Following an initial cycle of chemoimmunotherapy, during which his cryoglobulin levels increased from 5% to 35%, his treatment was revised to 4 weeks of weekly bortezomib (1.3 mg/m^2^), dexamethasone (40 mg/day), and rituximab (375 mg/m^2^), followed by 20 weeks of once weekly bortezomib (1.3 mg/m^2^) and dexamethasone (40 mg/day). The latter regime resulted in a good response that showed no detectable serum cryoglobulin at completion of chemotherapy.

Meanwhile, following on from satisfactory postoperative recovery, our patient underwent successful rehabilitation and was discharged from hospital.

## Discussion

WM is an extremely rare hematological cancer that may manifest with a spectrum of clinical features. Our patient presented with life-threatening acute bilateral lower limb ischemia, a toxic confusional state, elevated leukocyte count, and hyponatremia. Compared with a week previously, lower limb vascular assessment in this man was consistent with chronic smoking-related arteriopathy, which required lifestyle modification.

WM-associated acute vascular symptoms may arise from either cryoglobulinemia and/or hyperviscosity syndrome that is associated with increased production of abnormal paraproteins [7–9]. While cryoglobulinaemia is mostly known to result in distal arterial occlusive disease, hyperviscosity of serum is usually associated with IgM paraprotein concentrations greater than 50 g/L and significantly elevated serum viscosity (> 4.0 mPas), which can lead to vaso-occlusion [10, 11]. Accordingly, arterial occlusion of cardiac and cerebrovascular accidents have been reported in patients with WM [12, 13], but to the best of our knowledge, there have been no reports of acute occlusion of the popliteal arteries of the lower limb in the literature. Additionally, it is known that WM-associated neoplastic cells express procoagulant factors that interact with the vascular endothelium, red cells, and platelets, all of which promote thrombus formation [14]. Confounding factors for lower limb ischemia in our patient were his smoking habit and atrial fibrillation. While smoking is thrombogenic and may have contributed to his below knee arterial occlusion, atrial fibrillation characteristically causes distal vessel embolic occlusion, and is unlikely to have had an etiological role in acute arterial occlusion in this man. Acute bilateral occlusion of medium sized vessels of the lower limbs is rare, and could be attributed to a source from within the aorta or a more proximal site; transesophageal Doppler assessment excluded a cardiac source and CT scan did not show aortic pathology. Again, bilateral atrial fibrillation associated embolic phenomena in the lower limbs are likely to affect the small vessels in both feet, as is seen in “trash feet,” and do not usually result in ischemic occlusion of the infrapopliteal vessels. Thus, it seems logical to attribute bilateral below knee ischemia to hyperviscosity that was a feature of WM in this patient. Treatment of WM in this individual consisted of a combination of steroids, monoclonal antibodies, and the chemotherapeutic agent bendamustine that was later replaced by the targeted proteasome inhibitor bortezomib, which resulted in a satisfactory response.

## Conclusion

Acute lower limb ischemia, which is sudden reduction in lower limb arterial perfusion, is a major vascular emergency with an associated mortality of between 20% and 40%, and is limb threatening in 12–50% [15]. Acute and bilateral thrombotic occlusion of the lower limbs resulting from infrapopliteal ischemia in a patient with WM has not been previously reported in the literature. In this patient, emergency bilateral above-knee lower limb amputation was life saving.

## Data Availability

Not applicable.

## Abbreviations

WM: Waldenström’s macroglobulinaemia
IgM: Immunoglobulin M
CT: Computerized tomographic
MRI: Magnetic resonance imaging
